# Integration of metabolomics and transcriptomics identifies pathways involved in intermittent fasting and renal injury induced by a high-fat diet in mice

**DOI:** 10.3389/fphys.2025.1683573

**Published:** 2025-10-29

**Authors:** Lingfeng Yuan, Xiaobo Song, Yisheng Luan, Weihao Hong, Yue Hu, Shuqiao Ding, Bing Zhang, Yingzhe Xiong

**Affiliations:** ^1^ School of Physical Education and Sports, Central China Normal University, Wuhan, China; ^2^ Division of Sports Science and Physical Education, Tsinghua University, Beijing, China

**Keywords:** obesity, intermittent fasting, renal injury, metabolomics, transcriptomics

## Abstract

Obesity, a worldwide epidemic, is often accompanied by renal dysfunction or accelerating kidney disease. Intermittent fasting (IF) has become a popular weight loss approach, but the data for obesity-related kidney disease are very limited. Moreover, there is currently no combined omics study on its related metabolism, mechanisms, and pathways. The purpose of this study was to examine the preventive effect of IF on renal injury induced by a high-fat diet (HFD) and to explore the related pathways based on an omics analysis. We used an HFD to induce obesity-related renal injury. During IF intervention, the mice were allowed free access to regular chow every other day and were not provided food on the other day. Our result found that IF could effectively prevent obesity-related renal injury in glomerular morphological changes and urine components. Metabolomic and transcriptomic analyses revealed that IF affected the thermogenesis pathway, cholesterol metabolism pathway, and glycerolipid and glycerophospholipid metabolism pathways, and prevented and alleviated obesity-related renal injury through inflammation pathways and the insulin resistance pathway. This research would provide valuable data for the prevention and treatment of kidney diseases related to obesity.

## 1 Introduction

There is growing evidence that obesity is a worldwide health issue that poses significant risks to people’s lives and well-being. In addition, obesity is usually accompanied by complex metabolic diseases. Obesity-related renal injury is a type of glomerular injury due to obesity, which usually manifests as proteinuria and reduced renal function. Weisinger et al. (1974) first reported the phenomenon of proteinuria in severely obese patients in 1974. Other studies also have shown a strong association between obesity and kidney diseases (Camara et al., 2017; Martin-Taboada et al., 2021). Obesity leads to increased intraglomerular pressure and glomerular hypertrophy, which eventually causes glomerulosclerosis and functional impairment. If left uncontrolled and untreated, obesity-related renal injury may progress gradually, leading to chronic kidney disease and even end-stage renal disease. However, compared to diabetes or hypertension-related kidney disease, fewer studies focused on obesity-related kidney disease.

Lifestyle is one of the most important influencing factors for obesity, especially because the market is booming for high-calorie food and junk food. Concurrently, intermittent fasting (IF) is becoming increasingly popular for weight loss due to its convenience of not requiring individuals to count calories and its ability to improve metabolic diseases (Ezpeleta et al., 2024; Yang et al., 2022). Some studies have shown that IF also has potential ameliorative effects on kidney disease, especially autosomal polycystic kidney disease, through modulating the IGF-1 pathway, inhibiting the mTOR pathway, and promoting ketone body production (Chebib et al., 2024; Capelli et al., 2023). Some observational studies on patients with chronic kidney disease who fasted during Ramadan have been reported, suggesting that IF may help improve the estimated glomerular filtration rate and reduce proteinuria, but it may also lead to electrolyte imbalances and deterioration of kidney function based on individual differences (Baloglu et al., 2020; Malik et al., 2021). Therefore, the overall effect of IF on renal function remains unclear. Moreover, there are few studies that link IF and obesity-related kidney diseases, and even fewer studies that combine a multi-omics analysis of its related metabolism, mechanisms, and pathways.

In this study, we hypothesized that IF could effectively prevent obesity-related renal injury. First, we tested serum and urine components, along with glomerular morphological changes, to verify our hypothesis. Then, we used metabolomic and transcriptomic analyses to determine how IF affects the kidney in high-fat diet (HFD) mice. Our research would provide important data to related future studies and a valuable theoretical reference to the prevention and treatment of kidney diseases related to obesity.

## 2 Materials and methods

### 2.1 Animals and diets

Three-week-old male wild-type C57BL/6 mice were obtained and housed at the Laboratory Animal Research Center at Tsinghua University (Beijing, China). Following a 1-week acclimation period, the mice were randomly assigned to three groups (n = 7): a control (Con) group fed a chow diet containing 10% fat, 20% protein, and 70% carbohydrates (Beijing Keao Xieli Feed, China); a high-fat diet (HFD) group fed a diet containing 60% fat, 20% protein, and 20% carbohydrates (Beijing Keao Xieli Feed, China); and an intermittent fasting (HFD + IF) group also fed a high-fat diet. Mice in the Con and HFD groups had unrestricted access to food for 21 weeks. Mice in the HFD + IF group had unrestricted access to food for 13 weeks, and then, an 8-week IF intervention was initiated. During the 8-week IF intervention, the mice were allowed free access to regular chow every other day and were not provided food on the other day.

### 2.2 Biochemical parameters

At the end of the 21-week duration, body weights were measured, and urine was collected with abdominal pressure after a 12-h fast. Then, the mice were anesthetized, and blood samples were taken from the heart. The left kidneys were excised and stored in 4% paraformaldehyde for PAS staining. The right kidneys were snap-frozen in liquid nitrogen and stored at −80 °C for metabolomic and transcriptomic analyses. Serum was obtained by centrifuging the blood for 15 min at 3,000 rpm. Urine albumin, urine creatinine, serum glucose, and serum urea nitrogen (BUN) levels were determined using an automated chemistry analyzer (Kehua ZY KHB1280, China). Serum-free fatty acid (FFA) and serum cystatin-C (Cys-C) levels were analyzed using ELISA kits (MEIMIAN, China). PAS-stained sections were analyzed using a Panoramic DESK (3D Histech, Hungary) in conjunction with Image-Pro Plus 6.0 software. We measured the urine albumin-to-creatinine ratio (ACR), glomerular area (GA), and glomerular circumference (GC) for all mice.

### 2.3 Metabolomics detection

We extracted hydrophilic and hydrophobic compounds from the right kidney samples. For hydrophilic compounds, each 50 mg sample was combined with 1 mL of 70% methanol, and after centrifugation, 400 µL of the resulting supernatant was stored at −20 °C overnight. Subsequently, 200 µL of the supernatant was used for onboard analysis. For hydrophobic compounds, 20 mg of each sample was mixed with 1 mL of methyl-tert-butyl ether (MTBE), methanol, and an internal standard mixture. Then, 300 µL of the supernatant was extracted and concentrated after centrifugation. Kidney sample extracts were analyzed using an LC-ESI-MS/MS system (ExionLC AD UPLC-QTRAP, SCIEX, United States), along with Analyst 1.6.3 software (AB SCIEX, United States). Hydrophilic compounds were analyzed using a T3 UPLC C18 column and a UPLC amide column at 40 °C with a flow rate of 0.4 mL/min. Hydrophobic compounds were analyzed using a C30 column at 45 °C with a flow rate of 0.35 mL/min.

### 2.4 Transcriptomic detection

Total RNA was extracted from kidney tissues using TRIzol reagent (Invitrogen, United States) and further purified using an RNeasy Micro Kit (QIAGEN, Germany). cDNA libraries were constructed from the total RNA using the RNA-seq Library Prep Kit (NEB, United States) and verified using an Agilent 2100 TapeStation under standard protocols. Then, the cDNA libraries were sequenced on an Illumina NovaSeq 6000 platform. Clean reads were aligned to the reference transcriptome using HISAT2 (version 2.1.0), and differential gene expression analysis was conducted using DESeq2 (version 1.22.2).

### 2.5 Statistical analysis

For biochemical analyses, data are expressed as mean ± SD and were analyzed using one-way ANOVA followed by Tukey’s multiple comparison test in SPSS 25. A p-value <0.05 was considered statistically significant. Graphs were generated using GraphPad Prism 8.0. In omics analyses, we identified differentially abundant metabolites using the screening criteria fold change (FC) and variable importance in projection (VIP), criteria, specifically |log_2_(FC)| >1 and VIP >1. Differentially expressed genes were identified with P-values <0.01 and |log_2_(FC)| >1. The diagrams were generated using R software.

## 3 Results

### 3.1 IF prevented HFD-induced obesity and renal injury

**FIGURE 1:**
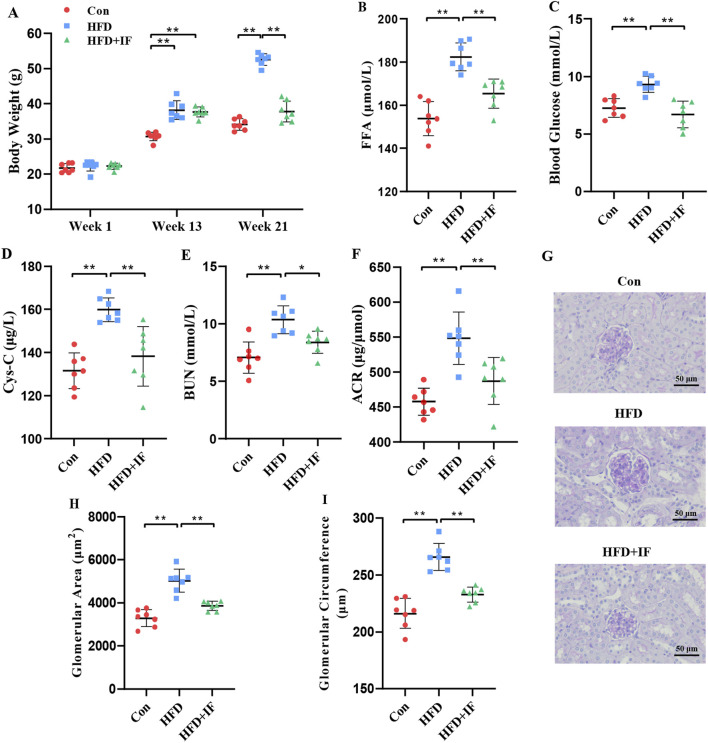
Assessment of obesity and renal function. **(A)** Body weight, **(B)** serum FFA, **(C)** serum glucose, **(D)** serum Cys-C, **(E)** serum BUN, **(F)** urine albumin-to-creatinine ratio, **(G)** PAS staining of the glomerulus (×400), **(H)** glomerular area, and **(I)** glomerular circumference. *p < 0.05; **p < 0.01.

To determine whether IF could effectively prevent obesity-related renal injury, we first used an HFD to induce obesity. Studies have shown that the seventh week marks a turning point in weight gain and that 10–12 weeks of HFD feeding can establish a stable obesity phenotype and associated metabolic characteristics (Buettner et al., 2007; Savetsky et al., 2015). According to our previous study, the HFD for 13 weeks could effectively induce obesity (Xiong et al., 2024), which was consistent with this study (Figure 1A). Next, we carried out IF intervention. At the end of 21 weeks, the HFD increased the levels of body weight, FFA, and blood glucose, but 8-week IF could prevent it (Figures 1A–C). Then, we measured serum Cys-C and BUN levels and urinary ACR to assess the condition of the kidney (Figures 1D–F) and found that 21 weeks of HFD feeding potentially induced renal injury, whereas IF intervention prevented it. GA and GC were evaluated using PAS staining (Figures 1G–I), revealing glomerular hypertrophy in HFD-fed mice, which was ameliorated by IF.

### 3.2 Metabolites involved in IF and HFD-induced renal injury

**FIGURE 2:**
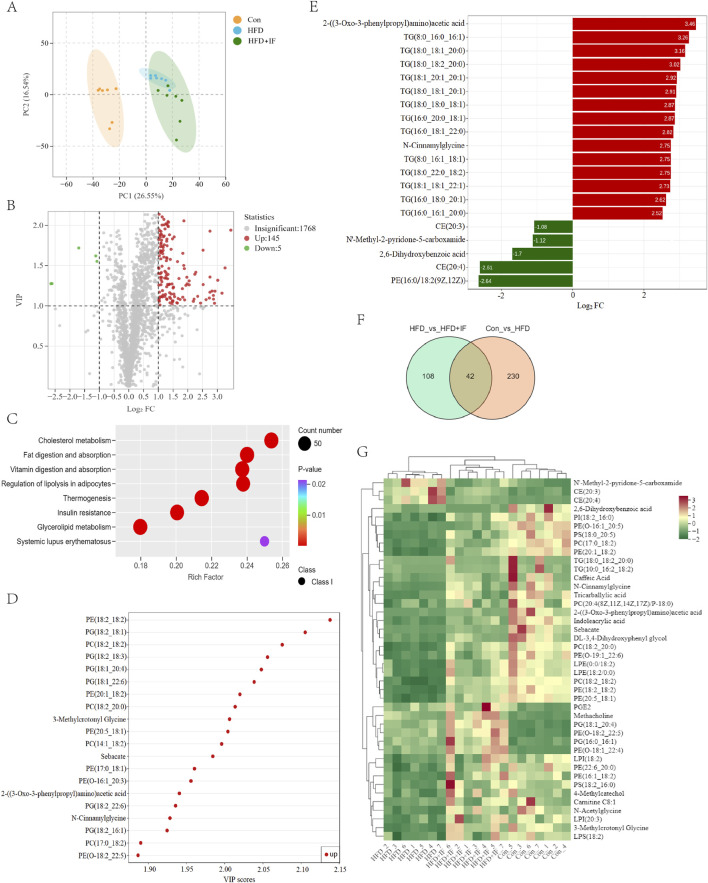
Metabolomics analysis. **(A)** PCA score plot between samples. **(B)** Volcano plot diagram of HFD vs. HFD + IF. **(C)** KEGG enrichment with P-value <0.05 pathways of HFD vs. HFD + IF. **(D)** The top 20 differentially abundant metabolites by VIP scores of HFD vs. HFD + IF. **(E)** The top 20 differentially abundant metabolites by log_2_(FC) of HFD vs. HFD + IF. **(F)** Venn diagram of HFD vs. HFD + IF and Con vs. HFD. **(G)** Clustering and heatmap visualization of 42 co-differentially abundant metabolites between samples.

To investigate metabolites involved in the improvement of obesity-related renal injury by IF, we used metabolomic analysis. The principal component analysis (PCA) showed a clear separation of our model group (Figure 2A). Through our screening process, we identified 150 differentially abundant metabolites, of which 145 were increased and 5 were decreased in the HFD + IF group compared to the HFD group (Figure 2B). The KEGG pathway analysis of these 150 metabolites revealed key pathways with P-values <0.05 (Figure 2C). The results showed that the main metabolic pathways included cholesterol metabolism, fat digestion and absorption, vitamin digestion and absorption, regulation of lipolysis in adipocytes, thermogenesis, insulin resistance, and glycerolipid metabolism.

In screening for differential metabolites, we found that the differentially abundant metabolites were mainly glycerolipids (GLs) and glyceryl phosphatides (GPs), such as TG, phosphatidyl ethanolamine (PE), phosphatidylglycerol (PG), and phosphatidylcholine (PC), based on the top 20 |log_2_(FC)| or VIP scores (Figures 2D,E). To identify the key metabolites, we compared differentially abundant metabolites between the Con group and the HFD group and between the Con group and the HFD + IF group, and found 42 co-differentially abundant metabolites, which were then clustered in each sample (Figures 2F,G). Among these 42 co-differentially abundant metabolites, we found that five metabolites were in the top 20 according to |log2 (FC)|: N′-methyl-2-pyridone-5-carboxamide, 2,6-dihydroxybenzoic acid, CE(20:3), CE(20:4), and TG(18:0_18:2_20:0). We also found nine metabolites in the top 20 according to VIP scores, namely, 3-methylcrotonyl glycine, sebacate, PG(18:1_20:4), PC(17:0_18:2), PC(18:2_20:0), PC(18:2_18:2), PE(20:1_18:2), PE(18:2_18:2), and PE(20:5_18:1). Moreover, we found that two metabolites were both in the top 20 according to |log_2_(FC)| and VIP scores: N-Cinnamylglycine and 2-((3-oxo-3-phenylpropyl) amino) acetic acid. These 16 key metabolites are presented in Table 1.

**TABLE 1:** Overview of 16 key co-differentially abundant metabolites.

	Compound	Class	Con vs. HFD	HFD vs. HFD + IF	KEGG map
N'-Methyl-2-pyridone-5-carboxamide	Heterocyclic compounds	Up	Down	ko00760 and ko01100
2,6-Dihydroxybenzoic acid	Organic acid and its derivatives	Down	Down	--
CE(20:3)	ST	Up	Down	ko00100, ko04913, ko04975, ko04976, ko04977, and ko04979
CE(20:4)	ST	Up	Down	ko00100, ko04913, ko04975, ko04976, ko04977, and ko04979
TG(18:0_18:2_20:0)	GL	Down	Up	ko00561, ko01100, ko04714, ko04923, ko04931, ko04975, ko04977, and ko04979
3-Methylcrotonyl glycine	Organic acid and its derivatives	Down	Up	--
Sebacate	Organic acid and its derivatives	Down	Up	--
PG(18:1_20:4)	GP	Up	Up	--
PC(17:0_18:2)	GP	Down	Up	ko00564, ko00590, ko00591, ko00592, ko01100, ko04723, and ko05231
PC(18:2_20:0)	GP	Down	Up	ko00564, ko00590, ko00591, ko00592, ko01100, ko04723, and ko05231
PC(18:2_18:2)	GP	Down	Up	ko00564, ko00590, ko00591, ko00592, ko01100, ko04723, and ko05231
PE(20:1_18:2)	GP	Down	Up	ko00563, ko00564, ko01100, ko04136, ko04140, ko04723, ko05130, and ko05167
PE(18:2_18:2)	GP	Down	Up	ko00563, ko00564, ko01100, ko04136, ko04140, ko04723, ko05130, and ko05167
PE(20:5_18:1)	GP	Down	Up	ko00563, ko00564, ko01100, ko04136, ko04140, ko04723, ko05130, and ko05167
N-Cinnamylglycine	Amino acid and its derivatives	Down	Up	--
2-((3-Oxo-3-phenylpropyl)amino)acetic acid	Amino acid and its derivatives	Down	Up	--

ST, sterol lipid; CE, cholesteryl ester; GL, glycerolipid; TG, triglyceride; GP, glycerophospholipid; PC, phosphatidylcholine; PG, phosphatidylglycerol; PE, Phosphatidylethanolamine. Con vs. HFD, up/down in HFD; HFD vs. HFD + IF, up/down in HFD + IF.

### 3.3 Genes involved in IF and HFD-induced renal injury

**FIGURE 3:**
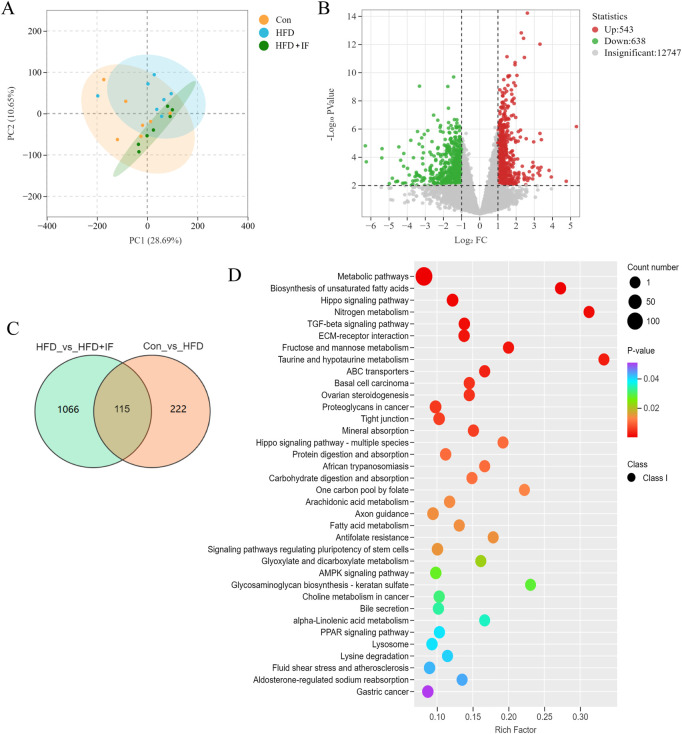
Transcriptomic analysis. **(A)** PCA score plot between samples. **(B)** Volcano plot diagram of HFD vs. HFD + IF. **(C)** Venn diagram of HFD vs. HFD + IF and Con vs. HFD. **(D)** KEGG enrichment with P-value <0.05 pathways of the differentially abundant metabolites of HFD vs. HFD + IF.

To explore the genes regulated by metabolites involved in IF-mediated attenuation of HFD-induced renal injury, we used transcriptome sequencing. The PCA score plot showed a clear separation of our model group (Figure 3A). Then, we identified 1,181 differentially expressed genes, of which 543 were increased and 638 were decreased in the HFD + IF group compared to the HFD group (Figure 3B). We analyzed the differentially expressed genes between the Con and HFD groups and between the HFD and HFD + IF groups, and found 115 co-differentially expressed genes (Figure 3C). We performed the KEGG pathway analysis for these 1,181 genes and displayed the pathways with P-values <0.05 (Figure 3D). We found that these pathways were not strongly related to glucolipid metabolism; rather, several pathways, such as the TGF-β signaling pathway and the ECM receptor interaction pathway, were closely related to renal injury. There was a slight difference between the results of metabolomic analysis and those of transcriptomic analysis.

### 3.4 Pathways involved in IF and HFD-induced renal injury

Then, correlation analysis of differential genes and differential metabolites was performed, and the results of Pearson correlation coefficients (PCCs) > 0.8 are shown in Table 2. There were 13 genes downregulated in Con vs. HFD and upregulated in HFD vs. HFD + IF, which strongly positively correlated with PC(18:2_20:0).

**TABLE 2:** Correlation analysis of differential genes and differential metabolites.

Con vs. HFD			HFD vs. HFD + IF		
Gene	Meta	PCC	Gene	Meta	PCC
Prodh	PC(18:2_20:0)	0.828	Prodh	PC(18:2_20:0)	0.828
Nit1		0.802	Nit1		0.802
Slc34a1		0.815	Slc34a1		0.815
Cyp2d26		0.804	Cyp2d26		0.804
Hpd		0.814	Hpd		0.814
Slc5a2		0.817	Slc5a2		0.817
Xylb		0.823	Xylb		0.823
Atxn7l2		0.825	Atxn7l2		0.825
Hsd3b2		0.837	Hsd3b2		0.837
Gm15441		0.821	Gm15441		0.821
Mroh7		0.866	Mroh7		0.866
G6pc		0.827	G6pc		0.827
Cyp2d12		0.826	Cyp2d12		0.826
Slc6a19		0.846	Syt3	Ile–Gly	0.81
Slc22a7		0.819	Ano3	Ile–Gly	0.839
Susd2	Carnitine ph-C1	0.811	Dcn	Guanidinoethyl sulfonate	−0.823
Car4	Carnitine ph-C1	0.824	Klhl13	Guanidinoethyl sulfonate	−0.805
Car4	3-Hydroxy-3-methylpentane-1,5-dioic acid	−0.825	Col3a1	Methacholine	−0.81

PC, phosphatidylcholine; red, upregulate; blue, downregulate; Con vs. HFD, up/down in HFD; HFD vs. HFD + IF, up/down in HFD + IF.

**FIGURE 4:**
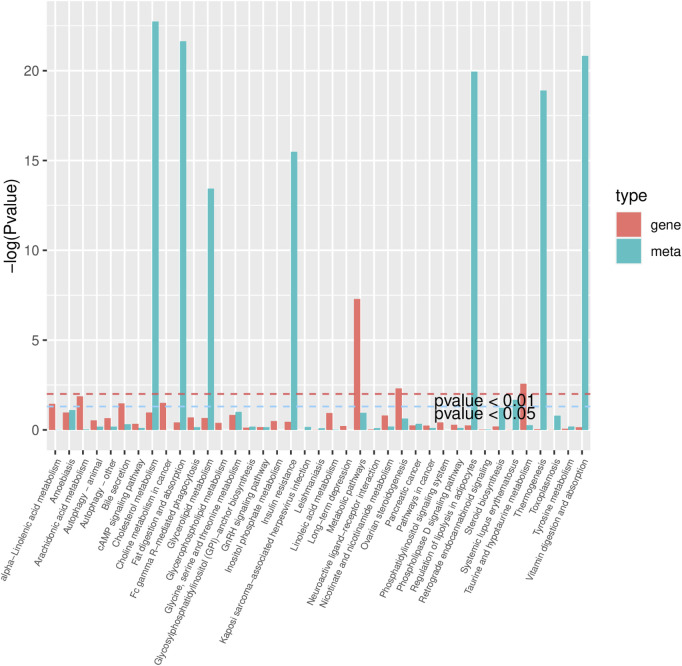
Pathways identified by both metabolomic and transcriptomic analyses.

To investigate the key biochemical pathways involved in the attenuation of HFD-induced renal injury by IF, we mainly focused on the differentially abundant metabolites and differentially expressed genes between the HFD group and the HFD + IF group. The co-pathways of these metabolites and genes are shown in Figure 4 and Table 3. The main pathways were thermogenesis, cholesterol metabolism, glycerolipid metabolism, insulin resistance, glycerophospholipid metabolism, autophagy–animal, arachidonic acid metabolism, and linoleic acid metabolism. These eight pathways involved at least six metabolites and five genes.

**TABLE 3:** Overview of pathways identified by both metabolomic and transcriptomic analyses.

	KEGG pathway	KEGG_id	Meta (n)	Gene (n)	Gene_name
Metabolic pathways	ko01100 ko04714	90	128	--
Thermogenesis		68	8	*Prkag3*, *Rps6kb2*, *Ndufb9*, *Uqcr11*, *Sos2*, *Ucp1*, *Cox8b*, and *Cox18*
Fat digestion and absorption	ko04975	67	3	*Acat3*, *Abca1*, and *Plpp3*
Cholesterol metabolism	ko04979	66	5	*Lpl*, *Lrpap1*, *Angptl4*, *Abca1*, and *Sort1*
Glycerolipid metabolism	ko00561	66	5	*Lpl*, *Tkfc*, *Akr1b8*, *Plpp3*, and *Mboat2*
Vitamin digestion and absorption	ko04977	66	1	*Slc19a1*
Insulin resistance	ko04931	64	7	*Prkag3*, *Rps6kb2*, *G6pc*, *Gys2*, *Ppargc1b*, *Mlxipl*, and *Pik3r3*
Regulation of lipolysis in adipocytes	ko04923	64	3	*Adora1*, *Npy1r*, and *Pik3r3*
Glycerophospholipid metabolism	ko00564	23	6	*Gpd1*, *Pla2g6*, *Pcyt2*, *Pla2g4a*, *Plpp3*, and *Mboat2*
Retrograde endocannabinoid signaling	ko04723	16	4	*Ndufb9*, *Gnao1*, *Plcb4*, and *Napepld*
Kaposi sarcoma-associated herpesvirus infection	ko05167	11	3	*Angpt2*, *Hif1a*, and *Pik3r3*
Autophagy–animal	ko04140	11	9	*Rps6kb2*, *Ambra1*, *Atg5*, *Atg10*, *Rraga*, *Hif1a*, *Pik3r3*, *Ulk2*, and *Uvrag*
Autophagy–other	ko04136	11	3	*Atg5*, *Atg10*, and *Ulk2*
Glycosylphosphatidylinositol (GPI)-anchor biosynthesis	ko00563	11	1	*Pgap1*
Arachidonic acid metabolism	ko00590	7	10	*Pla2g6*, *Cyp4a12a*, *Cyp4a14*, *Cyp2j13*, *Cyp2j9*, *Cyp2j6*, *Ggt1*, *Gpx3*, *Cbr2*, and *Pla2g4a*
Choline metabolism in cancer	ko05231	6	10	*Rps6kb2*, *Slc22a1*, *Slc22a5*, *Slc44a1*, *Sos2*, *Hif1a*, *Pdgfra*, *Plpp3*, *Pik3r3*, and *Pla2g4a*
Linoleic acid metabolism	ko00591	6	5	*Pla2g6*, *Cyp2j13*, *Cyp2j9*, *Cyp2j6*, and *Pla2g4a*
Systemic lupus erythematosus	ko05322	5	3	*C8g*, *H2az1*, and *H3f3a*
Amoebiasis	ko05146	5	9	*C8g*, *Lama3*, *Col3a1*, *Col4a5*, *Col1a1*, *Hspb1*, *Plcb4*, *Pik3r3*, and *Serpinb9*
Leishmaniasis	ko05140	5	1	*Cyba*
α-Linolenic acid metabolism	ko00592	5	4	*Pla2g6*, *Acaa1b*, *Acox3*, and *Pla2g4a*
Glycine, serine, and threonine metabolism	ko00260	5	4	*Shmt2*, *Alas2*, *Pipox*, and *Psat1*
Bile secretion	ko04976	2	10	*Slc22a1*, *Slc22a7*, *Slc22a8*, *Slc51b*, *Acnat2*, *Nr1h4*, *Aqp4*, *Atp1b3*, *Atp1b1*, and *Abcc4*
Ovarian steroidogenesis	ko04913	2	9	*Cyp2j6*, *Cyp2j9*, *Cyp2j13*, *Hsd3b4*, *Hsd3b2*, *Hsd17b2*, *Gm4450*, *Acot3*, and *Pla2g4a*
Phosphatidylinositol signaling system	ko04070	2	6	*Inpp1*, *Pik3c2a*, *Pik3c2b*, *Pik3r3*, *Ppip5k2*, and *Plcb4*
Steroid biosynthesis	ko00100	2	1	*Dhcr24*
Pancreatic cancer	ko05212	1	4	*Rps6kb2*, *Gadd45g*, *Pik3r3*, and *Tgfbr2*
Pathways in cancer	ko05200	1	28	*Rps6kb2*, *Mgst3*, *Bmp4*, *Gadd45g*, *Lama3*, *Zbtb16*, *Plekhg5*, *Axin2*, *Cdh1*, *Cdkn1b*, *Col4a5*, *Mecom*, *Vegfd*, *Fzd6*, *Fzd8*, *Lpar1*, *Hif1a*, *Pdgfra*, *Pik3r3*, *Plcb4*, *Ptch1*, *Ptger1*, *Shh*, *Sos2*, *Wnt5a*, *Wnt9b*, *Tgfbr2*, and *Rps6ka5*
Toxoplasmosis	ko05145	1	2	*Lama3* and *Gnao1*
GnRH signaling pathway	ko04912	1	4	*Mmp14*, *Pla2g4a*, *Plcb4*, and *Sos2*
Long-term depression	ko04730	1	3	*Gnao1*, *Pla2g4a*, and *Plcb4*
Fc gamma R-mediated phagocytosis	ko04666	1	7	*Pla2g6*, *Rps6kb2*, *Pik3r3*, *Pla2g4a*, *Plpp3*, *Ptprc*, and *Scin*
Neuroactive ligand–receptor interaction	ko04080	1	13	*Chrna4*, *Grik5*, *Pth1r*, *S1pr1*, *F2rl1*, *Calcrl*, *Ptger1*, *Pard3*, *Lepr*, *Adora1*, *Lpar1*, *Npy1r*, and *Prlr*
Phospholipase D signaling pathway	ko04072	1	8	*Dnm3*, *Lpar1*, *Pdgfra*, *Pik3r3*, *Pla2g4a*, *Plcb4*, *Plpp3*, and *Sos2*
cAMP signaling pathway	ko04024	1	12	*Acox3*, *Pde4d*, *Rap1a*, *Adora1*, *Atp1b3*, *Atp1b1*, *Npy1r*, *Pik3r3*, *Ptch1*, *Tiam1*, *Abcc4*, and *Hcar1*
Nicotinate and nicotinamide metabolism	ko00760	1	4	*Naprt*, *Sirt3*, *Aspdh*, and *Nadk2*
Inositol phosphate metabolism	ko00562	1	5	*Inpp1*, *Miox*, *Pik3c2a*, *Pik3c2b*, and *Plcb4*
Taurine and hypotaurine metabolism	ko00430	1	4	*Acnat2*, *Ggt1*, *Ggt6*, and *Ado*
Tyrosine metabolism	ko00350	1	1	*Hpd*

Red, upregulate; blue, downregulate. HFD vs. HFD + IF, up/down in HFD + IF.

## 4 Discussion and conclusion

IF is a new and popular weight loss strategy that can effectively regulate various health states in the body. In this study, we compared the effects of consistent HFD feeding with those of an HFD combined with IF on obesity-related renal injury, which is typically accompanied by glomerular hypertrophy and impaired glomerular filtration capacity (D'Agati et al., 2016). Therefore, we performed glomerular PAS staining to measure the GA and GC, and assessed urinary albumin and creatinine levels to calculate the ACR as key indices to confirm the morphological findings. The PAS staining results were consistent with the urine composition measurements. These findings suggest that HFD feeding induces obesity-related renal injury, whereas IF prevents and alleviates renal injury.

Kidney is a typical metabolic organ. To investigate the metabolites and genes involved in the improvement of obesity-related renal injury by IF, we performed comprehensive metabolomic and transcriptomic analyses, and explored the related pathways based on integrated omics approaches. We found that IF upregulated *Prkag3* and *Rps6kb2* and downregulated *Sos2*, *Ucp1*, and *Cox8b*, which are genes involved in the thermogenesis pathway. The *Prkag3* gene encodes the gamma3 subunit of AMPK (Crawford et al., 2010). *Rps6kb2* is regulated by the mTOR signaling pathway (Wu et al., 2022). *Sos2* is a key gene in the ERK signaling pathway and can inhibit the mTOR signaling pathway (Sudhakar et al., 2024; Dai et al., 2014). *Ucp1* and *Cox8b* are thermoregulatory genes (Garcia et al., 2016). A previous study revealed that UCP1 could alleviate lipid accumulation and suppress the development of acute kidney injury through the activation of the AMPK/ULK1/autophagy pathway (Xiong et al., 2021). However, recent evidence suggests that UCP-1 may not be present in the kidney (Sousa-Filho and Petrovic, 2025). Therefore, our findings regarding Ucp1 expression should be interpreted with caution and warrant further investigation. Another study revealed that changing the dietary composition could not promote adipocyte thermogenesis (Folie et al., 2022). However, our model involves IF, which changes feeding frequency rather than dietary composition. Therefore, the effects of IF on thermogenesis require further investigation. In addition, we identified 13 genes that were strongly positively correlated with PC(18:2_20:0), a type of phosphatidylcholine, which may serve as a key protective factor and biomarker. Previous studies have shown that decreased levels of PC are associated with reduced fatty acid oxidation capacity and lower muscle mass, and are closely linked to the mTOR pathway (Hsu et al., 2024). Our results found that IF could upregulate it. Taken together, we speculated that IF promoted the activation of the AMPK and mTOR signaling pathways, rather than renal cell thermogenesis, to alleviate renal injury.

Furthermore, IF upregulated *Angptl4*, *Lpl*, and *Lrpap1* and downregulated *Abca1* and *Sort1*, which are genes involved in the cholesterol metabolism pathway (Jensen et al., 2012). *Lpl* encodes an essential enzyme for triglyceride hydrolysis, and its activity is regulated by multiple factors, such as *Angptl4* (Spitler et al., 2021). *Lrpap1* encodes a receptor-associated protein (RAP) that negatively regulates low-density lipoprotein cholesterol levels. *Abca1* and *Sort1* are related to both high-density and low-density lipoprotein cholesterols. These findings might explain why IF could help with weight loss. In addition, a previous study revealed that RAP could protect the kidney from damage (Wagner et al., 2023), which might imply that the improvement in renal injury caused by IF was related to RAP.

Moreover, IF downregulated *Plpp3* and *Mboat2*, which are genes involved in the glycerolipid and glycerophospholipid metabolism pathways. *Plpp3* inactivates lysophosphatidic acid (LPA) to alleviate inflammation and thrombus formation (Aldi et al., 2018). *Mboat2* is related to arachidonic acid metabolism (Gijon et al., 2008), which is a lipid and immune pathway. Our research revealed that IF upregulated several genes in the CYP450 pathway involved in the linoleic acid metabolism, which is associated with the arachidonic acid metabolism. Studies have shown that promoting linoleic acid metabolism or arachidonic acid metabolism could effectively alleviate kidney inflammation (Wang et al., 2019; Chen et al., 2017). In addition, IF downregulated several important genes in the autophagy–animal pathway, such as *Rraga*, which encodes a subunit of Rag1/2, and *Atg5* and *Atg10*, which are components of ubiquitin-like conjugation systems. Studies have shown that IF and the kidney are strongly connected to autophagy in inflammatory responses (Mattson et al., 2017; Kimura et al., 2017). These findings indicate that IF alleviated renal injury through inflammatory pathways.

Regarding genes involved in insulin resistance pathways, IF upregulated *G6pc* and *Gys2,* which encode key enzymes involved in glucose homeostasis, and downregulated *Pik3r3*, which is a regulatory subunit of PI3K, to reduce glycogen production and increase the expression of gluconeogenic genes (Petersen and Shulman, 2018). In addition, IF upregulated *Ppargc1b*, which encodes PGC-1β, and *Mlxipl*, which encodes ChREBP, to promote lipogenesis (Witte et al., 2015). IF also upregulated *Prkag3* and *Rps6kb2*, which are related to the AMPK and mTOR signaling pathways (Crawford et al., 2010; Wu et al., 2022). Previous studies confirmed that increased insulin sensitivity could be beneficial to renal and vascular function (Artunc et al., 2016), and IF could effectively promote insulin sensitivity (Varady et al., 2021). Thus, we hypothesized that IF alleviated renal injury through insulin signaling.

Overall, our research revealed that IF affected the thermogenesis pathway, cholesterol metabolism pathway, and glycerolipid and glycerophospholipid metabolism pathways, and prevented and alleviated obesity-related renal injury through inflammation pathways and the insulin resistance pathway. However, this study has several limitations. First, we did not include a control group subjected to intermittent fasting (Con + IF), which limited our ability to evaluate the effects of IF under chow-diet conditions. The absence of this group indicates that we cannot fully distinguish whether the protective effects observed are specific to HFD-induced renal injury or whether IF itself has an impact on renal function in healthy mice. Future studies should incorporate the Con + IF group and use two-way ANOVA followed by Tukey’s multiple comparison test to comprehensively assess the independent and interactive effects of diet type and IF on renal injury. Second, we did not use RT-PCR or Western blot analysis to validate the expression of genes and proteins involved in the key pathways identified. Nevertheless, our data provide a valuable reference for related research in the future.

## Data Availability

The data presented in the study are deposited in the Figshare repository, available at https://figshare.com/articles/dataset/Dataset/30358597.
